# LHR-YOLO: A SAR small-target ship detection method based on improved YOLO11

**DOI:** 10.1371/journal.pone.0359334

**Published:** 2026-09-24

**Authors:** Miao Zhou, Dezhi Han, Xiang Shen, Yangshuyi Xu, Chongqing Chen

**Affiliations:** 1 School of Information Engineering, Shanghai Maritime University, Shanghai, China; 2 School of Computer Science, The University of Sydney, Sydney, New South Wales, Australia; Buckinghamshire New University - High Wycombe Campus: Buckinghamshire New University, UNITED KINGDOM OF GREAT BRITAIN AND NORTHERN IRELAND

## Abstract

Synthetic Aperture Radar (SAR) ship target detection holds significant application value in maritime traffic monitoring and marine environmental monitoring. However, due to challenges such as small ship targets, complex marine backgrounds, and speckle noise interference, existing methods still suffer from insufficient accuracy in small-target detection scenarios. To address this issue, this paper proposes a high-resolution detection algorithm named LHR-YOLO, based on YOLO11. In the Stem stage, a Gaussian–Laplacian edge-enhancement and Gaussian-filtering mechanism is introduced to effectively improve shallow-feature perception for tiny ship targets. In the Backbone, an improved C3k2 module is designed by incorporating channel shuffling, re-parameterized convolutions, and feature aggregation strategies to enhance multi-scale feature representation. Meanwhile, in the Neck–Detect stage, high-frequency perception, spatial dependency modeling, and a high-resolution detection head are integrated to further improve the localization accuracy of extremely small targets. Experimental results show that LHR-YOLO achieves ${mAP}_{50:95}$ values of 71.59% and 73.40% on the HRSID and SSDD datasets, respectively. Compared with YOLO11n, LHR-YOLO improves ${mAP}_{S}$ on HRSID from 53.95% to 60.53%, corresponding to an increase of 6.58 percentage points. These results validate the effectiveness of the proposed algorithm for detecting tiny ships in complex SAR maritime scenes and provide technical support for high-precision maritime target monitoring.The source code is available at: https://github.com/LRYTH/LHR-YOLO.

## Introduction

SAR forms a virtual long antenna through the motion of the radar platform and performs sophisticated processing of coherent echo signals, thereby achieving high-resolution two-dimensional imaging of ground scenes [1]. As an active microwave remote sensing system, SAR possesses all-weather, day-and-night observation capabilities and is highly sensitive to the physical properties of the Earth’s surface. Consequently, it holds irreplaceable application value in fields such as maritime surveillance and monitoring, disaster monitoring, ocean observation, and resource exploration [2,3].

Constant False Alarm Rate (CFAR), as a classic method in ship search and rescue as well as maritime target detection, has long been applied to ship detection tasks in radar and SAR images. This method achieves stable control of the false alarm rate to a certain extent by statistically modeling the local background and adaptively setting a detection threshold. However, under actual complex sea conditions, CFAR still exhibits significant limitations. On the one hand, it relies on idealized assumptions regarding the statistical characteristics of sea clutter, whereas the echoes from real sea surfaces exhibit pronounced non-stationarity, which can easily lead to detection threshold shifts [4]. On the other hand, in complex environments such as nearshore and harbor areas, the superposition of strong scatterers with the background can disrupt statistical consistency, thereby causing false alarms or missed detections. Furthermore, CFAR primarily makes decisions based on amplitude information and lacks the ability to model spatial structures and contextual features, resulting in limited detection performance for small-scale targets or targets with low signal-to-noise ratios [5]. Consequently, traditional CFAR methods struggle to meet the demands of high-precision detection in complex scenarios.

In recent years, with the advancement of deep learning, convolutional neural networks have been able to automatically learn discriminative features from complex backgrounds, demonstrating significant advantages in multi-scale target detection and sea clutter suppression. As a result, deep learning-based detection methods have gradually become mainstream [6]. Existing methods are primarily divided into two-stage and one-stage detection frameworks. Among them, two-stage methods generally achieve higher detection accuracy [7] but suffer from complex structures and high computational costs. In contrast, one-stage methods offer higher inference efficiency, making them more suitable for large-format SAR images and real-time monitoring scenarios.

Deep learning-based SAR ship detection methods have made certain progress. Zhao et al. [8] introduced large-receptive-field convolutions to mitigate speckle noise interference in SAR images, thereby improving the feature extraction process. Ke et al. [9] employed the Otsu thresholding method for sea-land segmentation, performing global thresholding on the grayscale distribution of images. In SAR images, ship targets often rely on clear edge contours and structural information for discrimination. However, existing methods typically employ standard convolutions for downsampling at the input stage, lacking an explicit modeling mechanism for high-frequency edge information. As a result, target structural details are weakened at the very beginning of feature extraction. Meanwhile, the presence of speckle noise further interferes with edge representation, limiting the discriminative capability of low-level features. Furthermore, current detection frameworks generally rely on FPN-like structures for multi-scale feature fusion. Selvam et al. [10] used an enhanced feature pyramid by adding a bottom-up augmentation path to the original FPN, while Zhang [11] introduced deformable convolutions into both the backbone network and the lateral connections of the FPN. However, most of these feature fusion methods are based on simple pixel-wise operations, lacking the ability to synergistically model high-frequency detail information and cross-layer spatial dependencies. In this process, the features of small-scale targets are gradually diluted or even overwhelmed through multiple transmissions, making it difficult to preserve their discriminative information, thereby compromising detection performance. In the feature extraction stage, Zhang et al. [12] achieved multiple and diverse interactions of features across different abstraction levels and functional modules through multi-path parallelization and cascading, yet the interaction mode remains singular. Existing methods have relatively limited mechanisms for interaction and fusion between different channels and different hierarchical features, making it difficult to fully exploit the potential correlations among features. This deficiency constrains the richness and discriminative power of feature representations, preventing the model from forming stable and distinctive feature representations in complex backgrounds.

The aforementioned issues commonly coexist in real-world SAR scenarios. Coastal and port areas contain sea clutter, islands, land structures, and other strong scatterers whose intensity and texture characteristics may resemble those of ships, increasing the risk of false alarms. Public datasets also exhibit a pronounced imbalance in ship scales. In the HRSID dataset, for example, small and medium-sized ships constitute the majority of samples, whereas large ships are comparatively scarce [13]. Because a small ship occupies only a limited area of the input image, repeated downsampling and projection into high-level feature spaces can weaken its spatial and discriminative information, resulting in localization errors and missed detections. Strong speckle noise further disrupts low-level texture and edge learning as well as feature interaction across levels. In nearshore areas, ships and coastal structures may both appear as bright scattering spots with blurred boundaries and unstable contrast, making stable target-contour learning particularly difficult [14]. Collectively, these factors constrain detection accuracy and robustness in complex SAR scenes and motivate more effective preservation and fusion of small-target information.

To address the aforementioned problems and inspired by studies [15–17], this paper proposes an improved framework based on YOLO11 for SAR tiny ship target detection. First, an Edge-aware Enhancement Stem (EES-Stem) is designed, which introduces Gaussian–Laplacian edge enhancement and Gaussian smoothing constraints during downsampling to improve the representation capability of shallow structural details. Second, a Frequency-Spatial Collaborative Feature Pyramid Network (FSC-FPN) is proposed, which integrates high-frequency perception and cross-layer spatial dependency modeling to strengthen the discriminative information retention capability of small targets during multi-scale propagation. Furthermore, a Reparameterized Shuffle Aggregation Block (RSA-Block) is designed, incorporating structural reparameterization, channel interaction, and multi-stage aggregation to enhance cross-layer feature interaction and robust representation capability under complex backgrounds.

The main contributions of this paper are as follows:

1. An edge-aware improved Stem structure, termed EES-Stem, is proposed to enhance the structural perception capability of shallow tiny targets through a synergistic mechanism of edge enhancement and denoising.
2. A high-frequency and spatial dependency collaborative modeling method, termed FSC-FPN, is introduced to achieve joint modeling of high-frequency details and spatial dependencies, thereby improving the cross-layer representation capability of small targets.
3. An RSA module is designed to effectively integrate channel shuffling, structural reparameterization, and multi-stage aggregation, enhancing feature discriminative capability under complex backgrounds while maintaining computational efficiency.

The remainder of this paper is organized as follows. The Related Work section reviews recent advances in SAR image target detection. The Method section introduces the relevant technologies of YOLO11 and presents the proposed LHR-YOLO model. The Experimental results and analysis section describes the datasets and evaluation metrics, evaluates the proposed model through ablation and comparative experiments, and analyzes scene-specific false-positive and false-negative behavior. Finally, the Conclusion section summarizes the main findings and outlines future research directions.

### Related work

In recent years, researchers have proposed various algorithms to address challenges in SAR image target detection, such as image noise interference, blurred target features, and detection issues related to small targets. From traditional methods to deep learning-based detectors, each approach has focused on improving detection accuracy and robustness.

### Traditional SAR ship detection

Based on the content of Section 1, CFAR method played an important role before the rise of deep learning. In the field of SAR image target detection, CFAR and its variants remain representative of traditional methods. Early research primarily focused on accurately modeling the statistical characteristics of sea clutter to improve detection performance. For example, Hamidi [18] modeled sea clutter based on the Weibull distribution and designed an adaptive threshold detector, but its performance depends on the degree of match between the model and the actual scene. To enhance adaptability to non-homogeneous clutter, Meng [19] proposed a non-parametric CFAR method based on the Wilcoxon rank-sum test, which avoids reliance on distributional assumptions but suffers from limited performance in small-target or edge-region detection. To address false alarm issues in complex scenes, Pappas et al. [20] extended CFAR to the superpixel level to preserve structural information, yet its effectiveness depends on segmentation quality. Furthermore, Leng et al. [21] improved background estimation through a bilateral scanning strategy, thereby enhancing detection performance. Although these CFAR methods are effective to a certain extent, they still exhibit significant limitations in complex sea clutter and small-target scenarios. In contrast to CFAR methods that rely on statistical modeling and amplitude-based decision making, this paper enhances target structural representation and mitigates the loss of small-target features during transmission through explicit edge information modeling and a multi-scale feature synergistic mechanism, thereby achieving more stable detection performance in complex sea clutter environments.

### Deep learning-based detection algorithms

To address the performance bottlenecks and challenges faced by traditional ship detection methods under complex backgrounds and multi-scale target conditions, convolutional neural network (CNN)-based ship detection techniques have been extensively studied and have achieved remarkable progress in the field of SAR image analysis in recent years. From the perspective of the detection pipeline, existing methods are mainly divided into two-stage detectors and one-stage detectors.

### Two-stage detection

Two-stage object detection methods typically first generate a set of candidate regions and then perform fine-grained classification and bounding box regression on these regions, thereby achieving high detection accuracy. Representative algorithms include Fast R-CNN [22], Faster R-CNN [23], and Cascade R-CNN [24], which have achieved satisfactory performance in general object detection tasks. However, these methods still exhibit certain limitations in SAR ship detection scenarios. First, the candidate region generation stage lacks sufficient adaptability to small-scale ship targets, which tends to reduce the recall rate and cause tiny targets to be missed in the first stage. Second, these methods require generating and processing a large number of candidate regions, resulting in high computational complexity and limited inference speed, making it difficult to meet the real-time application demands of high-resolution or large-format maritime scenes. Furthermore, the ubiquitous speckle noise and complex sea clutter in SAR images easily interfere with the candidate region generation process, increasing the risk of false positives. In terms of multi-scale modeling, the scale of candidate regions is constrained by the anchor design and feature hierarchy, leading to insufficient adaptability to ship targets with significant scale variations or low signal-to-noise ratios. Meanwhile, the overall pipeline of these methods is relatively complex, involving numerous hyperparameters that rely on empirical tuning, which reduces the algorithmic generality and engineering practicality.

To address the aforementioned issues, some studies have improved the two-stage detection framework from the perspective of feature modeling. For example, one study introduced a scattering region topological structure pyramid (SRTP) to fuse global contextual information and structural difference features, thereby enhancing the discriminative capability between ships and backgrounds and reducing false positives while improving multi-scale detection performance to a certain extent [25]. However, its characterization of fine-grained features in complex backgrounds remains insufficient. On the other hand, the combination of an adaptive threshold-based STQM and a shallow-deep feature fusion module (LGCFM) can effectively suppress background noise and improve small-target detection performance [26]. DSF-Net enhances small-target detection through multi-domain feature mixing [27], yet there is still room for improvement in high-frequency detail information modeling. In contrast to the above methods, this paper reinforces low-level fine-grained structural representation and mitigates the attenuation of small-target features during cross-layer transmission through explicit edge information modeling and a high-frequency-spatial collaborative feature fusion mechanism, while enhancing feature representation capability via channel interaction, thereby achieving more stable and discriminative detection performance under complex backgrounds.

Overall, although the above methods have improved the performance of the two-stage detection framework to a certain extent, they still face limitations such as constrained inference efficiency and insufficient representation of small-scale targets and detailed features, which restrict their application in real-time SAR ship detection tasks.

### One-stage detection

As a typical one-stage detection framework, the YOLO series achieves object classification and bounding box regression through an end-to-end convolutional neural network, delivering high detection accuracy while maintaining favorable computational efficiency. Consequently, it has been widely adopted in real-time object detection tasks. To address the challenges of limited small-target scales, weak features, and susceptibility to background interference in remote sensing images, existing studies have made various improvements to the YOLO framework in terms of feature enhancement and multi-scale modeling.

In terms of small-target detection and feature fusion, SCAF-YOLO enhances global semantic representation by introducing a spatial context-aware module and an adaptive feature fusion mechanism [28]. SOD-YOLO improves small-target detection capability by adding a high-resolution detection layer and incorporating a multiscale fusion strategy [29]. LRDS-YOLO adopts lightweight downsampling and an improved feature pyramid structure to enhance small-target representation under complex backgrounds [30]. SMA-YOLO and LKPF-YOLO mitigate false positives and feature missing issues from the perspectives of feature fusion and convolution modeling, respectively [31,32]. However, the above methods primarily focus on semantic-level enhancement or structural-level improvements, lacking explicit modeling of high-frequency details and edge information in low-level features. Consequently, they are susceptible to speckle noise interference in SAR images, leading to unstable target structural representation. To address this issue, this paper introduces EES-Stem at the input stage, which explicitly enhances high-frequency structural information through an edge-aware mechanism, thereby improving the stability and discriminative capability of feature representation from the very beginning.

Furthermore, in terms of complex background and multiscale feature interaction, YOLO-PFA utilizes a bidirectional feature pyramid structure to enhance cross-scale information interaction [33]. YOLO-Lite combines a lightweight backbone network with a context fusion module to achieve a certain balance between efficiency and accuracy [34]. Although the above methods have achieved some success in cross-scale fusion and feature enhancement, their feature fusion processes mostly rely on implicit modeling approaches, lacking a collaborative characterization of high-frequency information and spatial dependencies. As a result, small-target features remain prone to attenuation during multi-layer transmission. Meanwhile, the feature interaction mechanisms across channels and between levels remain insufficient, limiting the richness of feature representation. To address these deficiencies, this paper proposes FSC-FPN at the feature fusion stage, which achieves synergistic transmission of fine-grained information and semantic information through high-frequency perception and cross-layer spatial dependency modeling. Furthermore, an RSA module is designed to enhance feature interaction capability via channel shuffling and multi-stage feature aggregation, thereby improving the model’s representation capability under complex scenarios.

In summary, although existing methods have made certain progress in small-target detection, multiscale fusion, and complex background modeling, they still suffer from deficiencies in edge structure modeling, high-frequency information preservation, and feature interaction mechanisms. This paper systematically optimizes the aforementioned issues through targeted improvements at three levels: input, fusion, and feature modeling. Unlike approaches that address these limitations separately, LHR-YOLO combines explicit edge-aware input processing, frequency-spatial cross-scale fusion, and reparameterized feature aggregation within a unified YOLO11-based detector for SAR small-ship detection.

## Method

This chapter first introduces the basic structure and characteristics of the baseline model YOLO11, then presents the improved model proposed in this paper, LHR-YOLO, and elaborates on its innovations.

### YOLO11

The YOLO series adopts a one-stage object detection paradigm, integrating object localization and category prediction into a unified network. That is, it directly outputs the bounding boxes and category information of targets from the input image without requiring additional candidate region generation or complex intermediate processing steps. This end-to-end detection framework significantly reduces computational overhead while improving overall inference efficiency. Compared with traditional two-stage detection methods, the YOLO series achieves faster detection speed while maintaining high detection accuracy.

**Fig 1:**
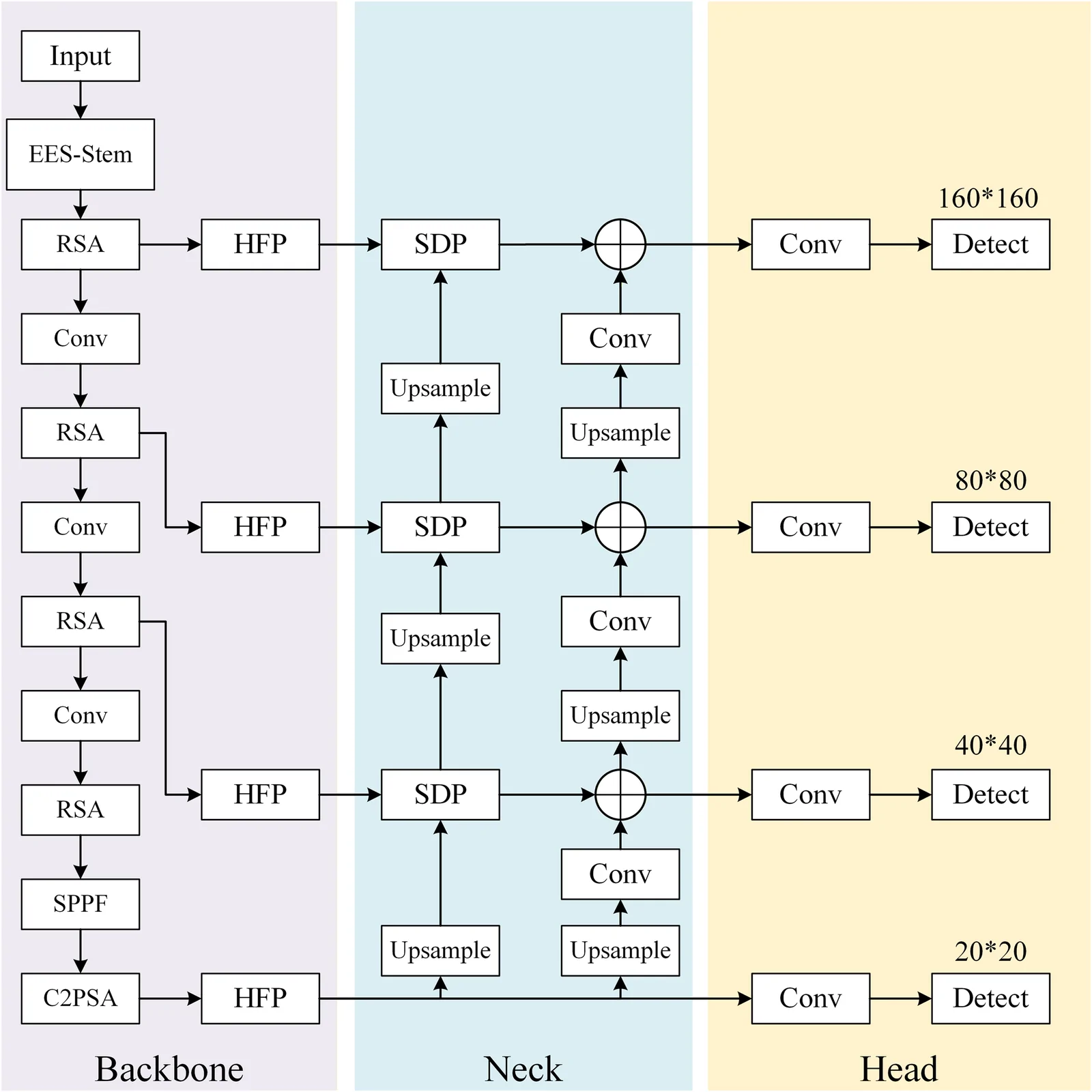
Architecture of the YOLO11 model. The marked components (dashed boxes and colored regions) correspond to the main improvements proposed in this paper.

The baseline model YOLO11 is a relatively recent object detection model in the YOLO series. Compared with YOLOv8, it incorporates further structural optimizations and performance improvements in key modules such as the Backbone, Neck, and Detect Head, demonstrating superior detection accuracy and inference efficiency across various computer vision object detection tasks. Therefore, this paper selects YOLO11 as the baseline model and makes improvements and extensions upon it. The architecture of the YOLO11 model is shown in Fig 1.

From an overall architectural perspective, YOLO11 adopts a typical one-stage detection framework, consisting mainly of three components: Backbone, Neck, and Detect Head. The Backbone network is responsible for extracting multi-level semantic features from the input image. By introducing efficient convolutional structures and attention mechanisms, it effectively enhances the modeling capability of key target features. The Neck module further fuses and enhances features from different levels, achieving multiscale feature fusion through operations such as upsampling and feature concatenation, thereby improving the network’s representation capability for targets at different scales. The Detect Head performs parallel prediction based on the fused multiscale feature maps, outputting category information and location information of targets, demonstrating good adaptability particularly in detecting both small and large targets. YOLO11 has three Detect Heads, each operating on feature maps at different scales (shallow, middle, and deep feature layers). The shallow head is responsible for detecting small targets, while deeper layers are used to detect larger-scale objects. In the Neck module responsible for feature fusion, information is progressively transmitted from shallow to deep layers for multiscale fusion, which may lead to the loss of critical information, such as that of small targets, thereby affecting object detection performance. To address this issue, this paper makes improvements based on certain structures of YOLO11, enhancing shallow features and reducing the loss of small-target information during feature fusion, thus improving the model’s performance in detection tasks.

### LHR-YOLO

**Fig 2:**
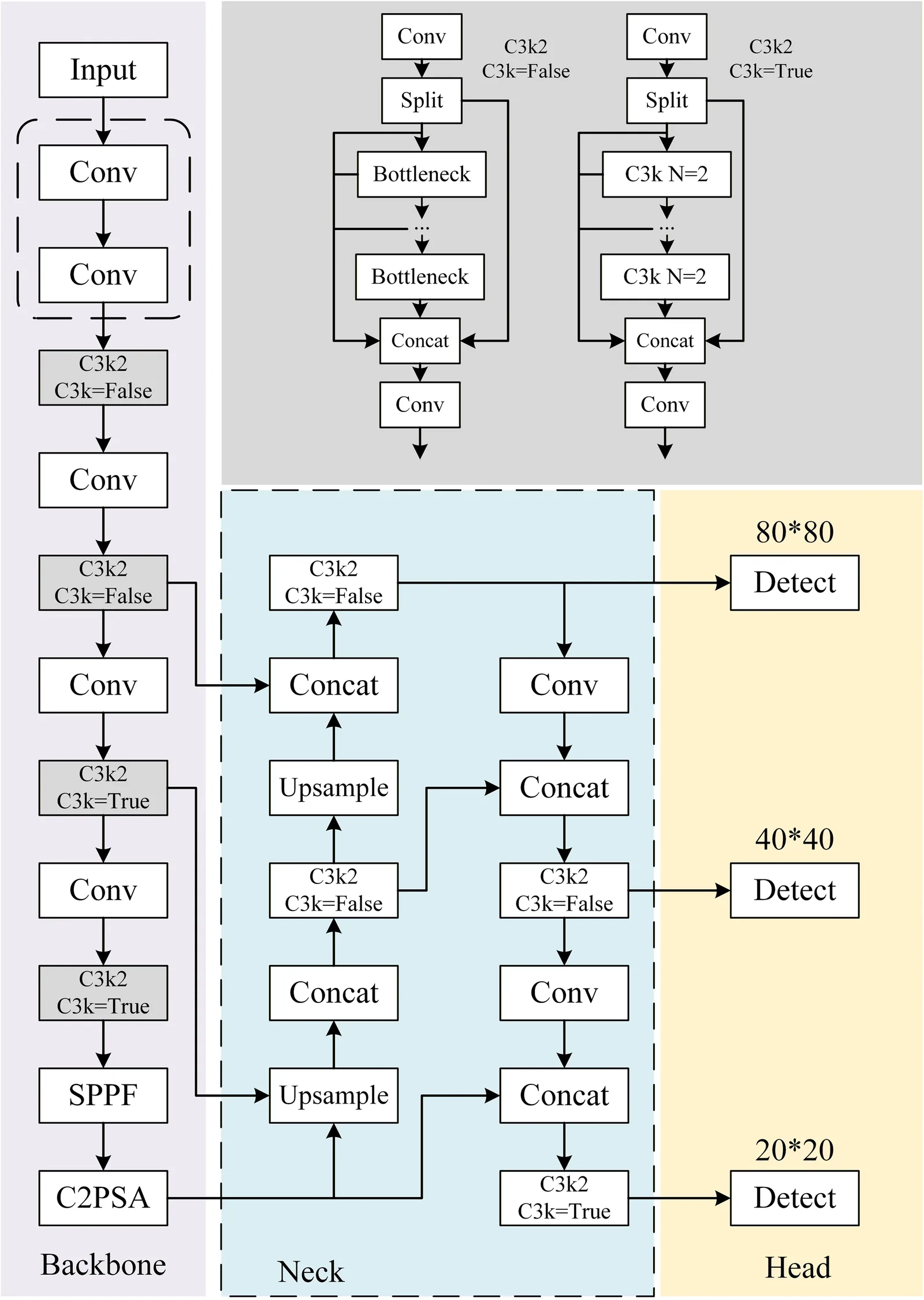
Architecture of the LHR-YOLO model.

To address the common issues in SAR images, such as edge structure degradation, the vulnerability of small-target features to attenuation during multiscale transmission, and insufficient feature interaction, this paper systematically improves the YOLO11 framework from the perspective of feature modeling. First, to tackle the problems of blurred target boundaries and ineffective extraction of high-frequency structural information caused by speckle noise, an edge-aware improved Stem structure, termed EES-Stem, is designed at the input stage. This structure explicitly models image edge information by introducing a Laplacian of Gaussian operator, while suppressing noise through Gaussian smoothing constraints, thereby enhancing structural details and discriminative capability in low-level features. Second, to address the issue that small-target features are gradually attenuated or even lost due to multiple downsampling and cross-layer transmission in the feature pyramid, the Neck structure is improved. A Frequency-Spatial Collaborative FPN (FSC-FPN) is proposed, which introduces a high-frequency perception mechanism in the lateral connections to strengthen fine-grained information expression. Furthermore, cross-layer pixel-level dependency modeling is incorporated to promote effective fusion of semantic information and detail information, thereby improving the feature preservation capability for small targets. Finally, to overcome the limitations of insufficient interaction across feature channels and between levels, as well as constrained feature representation capability in existing methods, an RSA module is designed. It enhances information flow among features through channel splitting and channel shuffling strategies, and further improves feature representation capability via multi-stage feature aggregation. As a result, the model is able to form more stable and discriminative feature representations under complex backgrounds. The overall framework of the LHR-YOLO model is illustrated in Fig 2.

### EES-Stem

**Fig 3:**
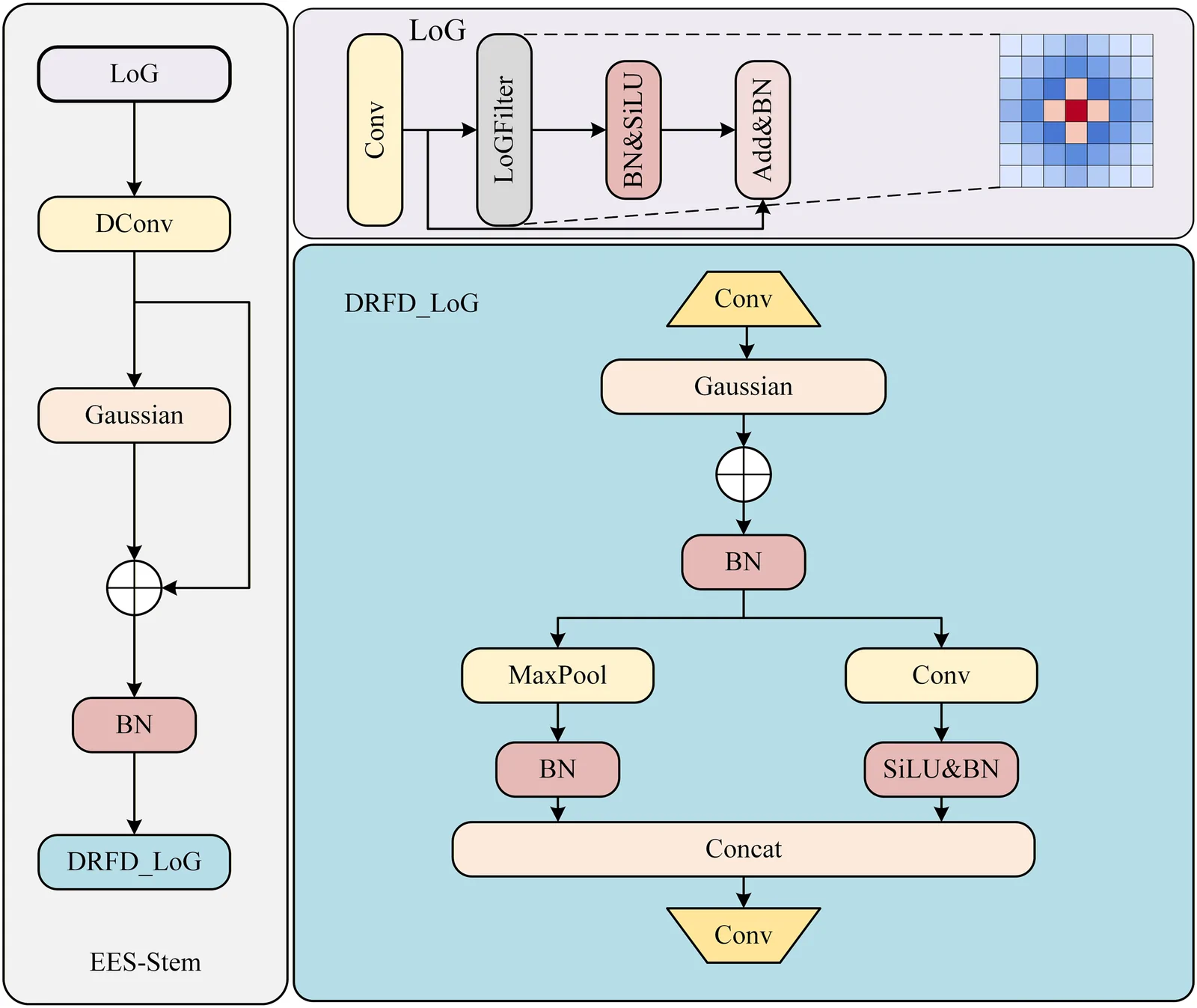
Architecture of the EES-Stem module.

In the initial feature extraction stage of the network, a Laplacian of Gaussian (LoG) operator is introduced to effectively capture the edge features of targets while performing initial downsampling. The LoG operator is a commonly used edge detection operator that combines Gaussian smoothing with the Laplacian operator to achieve effective extraction of image edges and fine structural details. It consists of a Gaussian filter and a second-order Laplacian differential operator. Its principle is to first smooth the image via Gaussian filtering to suppress noise interference, and then apply the second-order Laplacian operator to the smoothed image, thereby highlighting regions with sharp grayscale variations, i.e., target edges and contours. Due to these dual functionalities, the LoG operator exhibits good applicability in SAR images characterized by high noise levels and strong background interference. The structural diagram of the EES-Stem module is shown in Fig 3.

Assume the input image $I\in {\mathbb{R}}^{H\times W\times 3}$. It first undergoes preliminary feature extraction through a 7×7 convolution, followed by a LoG filter with a kernel size of 7×7 and σ=1.0, enabling the network to learn feature representations and thereby emphasize target edges. The definitions of the two-dimensional LoG filter and Gaussian filter at position (*x*,*y*) are as follows:


$$\begin{array}{c} {\mathrm{LoG}}_{\sigma }^{k\times k}(x,y)=\frac{1}{\pi \sigma^{4}}\left(1-\frac{x^{2}+y^{2}}{\sigma^{2}}\right)\exp\left(-\frac{x^{2}+y^{2}}{2\sigma^{2}}\right) \end{array}$$ (1)

$$\begin{array}{c} {\mathrm{Gaussian}}_{\sigma }^{k\times k}(x,y)=\frac{1}{2\pi \sigma^{2}}\exp\left(-\frac{x^{2}+y^{2}}{2\sigma^{2}}\right) \end{array}$$ (2)

where k×k denotes the kernel size and σ denotes the standard deviation. In our implementation, the LoG and Gaussian-filter parameters were empirically set to a 7×7 kernel with σ=1.0 and a 9×9 kernel with σ=0.5, respectively, and kept fixed throughout all experiments.

The feature *I*_1_ obtained after the 7×7 convolution is enhanced through a residual connection. This connection first passes the output of the LoG filter through an activation function and batch normalization to obtain $F_{LoG}$:


$$\begin{array}{c} F_{LoG}=Norm\;\left(I_{1}+ACT\;\left({\mathrm{LoG}}_{1.0}^{7\times 7}\;\left(I_{1}\right)\right)\right) \end{array}$$ (3)

Here, Norm(·) denotes batch normalization and ACT(·) denotes the SiLU activation function. In the following equations, Conv(·) denotes a standard convolution, ConvD(·) denotes a downsampling convolution, and Concat(·) denotes channel-wise concatenation.

Subsequently, $F_{LoG}$ undergoes another downsampling operation to obtain *F*_1_, which reduces the spatial dimensions to $\frac{H}{2}\times \frac{W}{2}$ while preserving edge and spatial structural features through gentle downsampling:


$$\begin{array}{c} F_{1}={ConvD}_{3\times 3}\;\left({Conv}_{3\times 3}\;\left(F_{LoG}\right)\right) \end{array}$$ (4)

*F*_1_ undergoes Gaussian smoothing and residual fusion. A Gaussian filter with a kernel size of 9×9 and σ=0.5 is applied to *F*_1_ to perform smoothing, thereby suppressing the interference of noise on edge information. The smoothed features are then residually fused with the original features of *F*_1_ and normalized via batch normalization (BN) to obtain *F*_2_:


$$\begin{array}{c} F_{2}=Norm\;\left(F_{1}+{\mathrm{Gaussian}}_{0.5}^{9\times 9}\;\left(F_{1}\right)\right) \end{array}$$ (5)

Through this operation, the stability of low-frequency features is enhanced while preserving edge and contour information, thereby preparing the features for subsequent multiscale fusion. *F*_2_ is then fed into the DRFD module. Within this module, features are processed through two parallel paths: a convolution path (Depthwise Conv + BN + activation) to enhance feature representation capability, and a max-pooling path (MaxPool + BN) to retain the most salient responses in the feature map. Additionally, a Gaussian filter is incorporated within the module to further smooth the features and integrate multiscale uncertainty information. The outputs of the two paths are concatenated along the channel dimension and then fused through a 1×1 convolution to obtain the final downsampled feature map $F_{stem}$:


$$\begin{array}{c} F_{stem}=DRFD\;\left(F_{2}\right) \end{array}$$ (6)

After passing through the DRFD module, the spatial dimensions of the feature map are reduced to $\frac{H}{4}\times \frac{W}{4}$, while simultaneously preserving edge, texture, and low-frequency information.

### FSC-FPN

**Fig 4:**
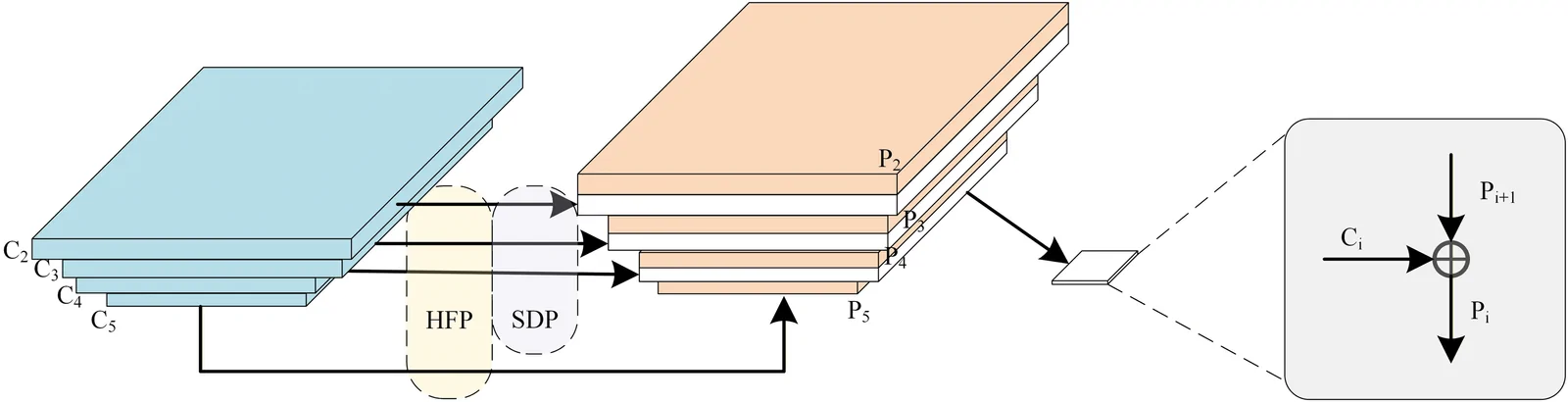
Architecture of the FSC-FPN.

This paper introduces targeted improvements to the Neck component of the YOLO model. In the constructed multiscale feature fusion framework, feature maps at different levels are extracted from the backbone network, and a dual-branch enhancement mechanism is incorporated into the lateral fusion paths. Specifically, each lateral connection integrates two key processes: high-frequency information enhancement and cross-layer relationship modeling. Among them, the High Frequency Perception (HFP) mechanism effectively highlights the structural and edge information of tiny targets by strengthening the high-frequency responses in the feature maps. Meanwhile, a spatial dependency modeling strategy based on pixel-level interaction characterizes the correspondences between features of adjacent layers, enabling high-level semantic information to more effectively guide low-level feature expression, thereby improving the representation capability of small targets. In terms of structural design, the high-frequency enhancement process is applied to all feature fusion paths across scales, while Spatial Dependency Modeling (SDP) is primarily applied to the higher-resolution layers (*P*_2_, *P*_3_ and *P*_4_). Finally, the multiscale features are integrated through independent convolution operations and fed into the detection heads for target prediction. The overall architecture of FSC-FPN is shown in Fig 4.

(1) **High Frequency Perception Module (HFP)**

**Fig 5:**
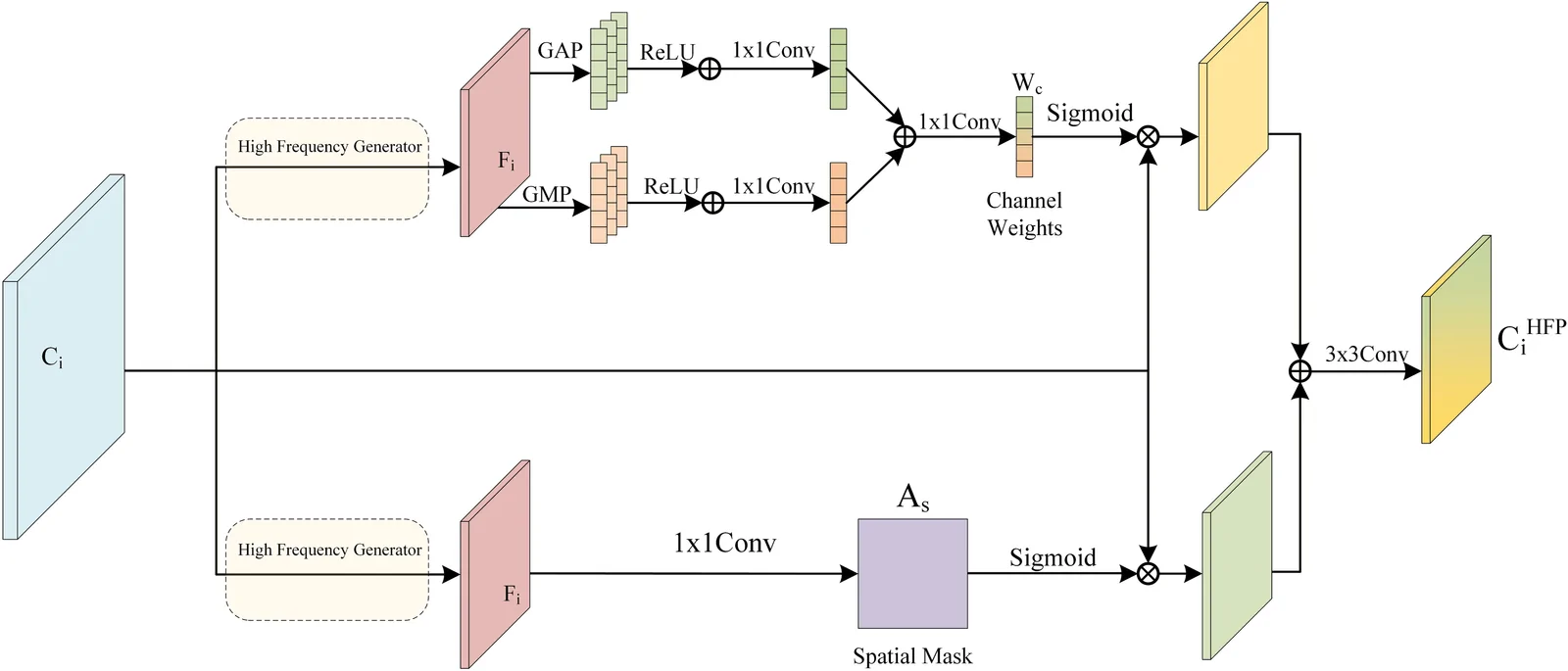
Architecture of the high frequency perception module.

In traditional feature pyramid structures, the feature fusion process lacks explicit modeling of tiny targets, which can easily lead to their discriminative information being overwhelmed by the background. To address this issue, this paper designs a High Frequency Perception Module (HFP) to enhance the high-frequency information related to small targets in the feature map. Its overall architecture is shown in Fig 5. Let the input feature map be denoted as $C_{i}\in {\mathbb{R}}^{C\times H\times W}$.

First, a Discrete Cosine Transform (DCT) is applied to the input feature map to convert it from the spatial domain to the frequency domain, thereby obtaining its corresponding spectral representation. Based on the characteristic that low-frequency components are concentrated in the central region of the spectrum while high-frequency components are distributed at the edges, this paper designs a high-pass filtering strategy that introduces adjustable parameters to suppress the low-frequency region, thereby reducing the influence of global smoothing information. On this basis, the high-frequency components are retained and enhanced, enabling the network to focus more on small targets and their local detail features. Finally, an Inverse Discrete Cosine Transform (iDCT) is applied to map the processed frequency-domain features back to the spatial domain, yielding the enhanced feature representation $F_{i}$. Hereafter, DCT(·) and iDCT(·) denote the forward and inverse two-dimensional discrete cosine transforms, respectively. This process effectively improves feature representation capability and enhances the model’s perception of fine-grained details under complex backgrounds without introducing additional complex structures.

Subsequently, for the enhanced feature $F_{i}$, on one hand, through global statistical modeling of the high-frequency features, $F_{i}$ is first processed by global max pooling and global average pooling. The results are then passed through an activation function and summed along the spatial dimension to generate two one-dimensional channel description vectors. These two vectors are then mapped to channel weights via 1×1 convolutions and fused to generate the channel weight $W_{c}$, which characterizes the importance of different channels in tiny target representation. On the other hand, a spatial attention distribution $A_{s}$ is constructed using the high-frequency responses to guide the network to focus on potential target regions. Compared with direct modeling based on raw features, this strategy effectively reduces low-frequency background interference, making the attention allocation more biased toward discriminative local regions.

Finally, the channel-modulated and spatial-modulated features are fused, and the integrated features are obtained through a convolution operation to produce the output feature $C_{i}^{HFP}$:


$$\begin{array}{c} C_{i}^{HFP}=Conv\;\left(C_{i}⊙\sigma \;\left(W_{c}\right)+C_{i}⊙\sigma \;\left(A_{s}\right)\right) \end{array}$$ (7)

In this expression, σ(·) is the sigmoid function and ⊙ denotes element-wise multiplication.

By introducing a high-frequency information guidance mechanism, the HFP module is able to enhance the structural representation of tiny targets under complex background and noise interference conditions, thereby improving the effectiveness of the overall feature representation.

(2) **Spatial Dependency Perception Module (SDP)**

**Fig 6:**
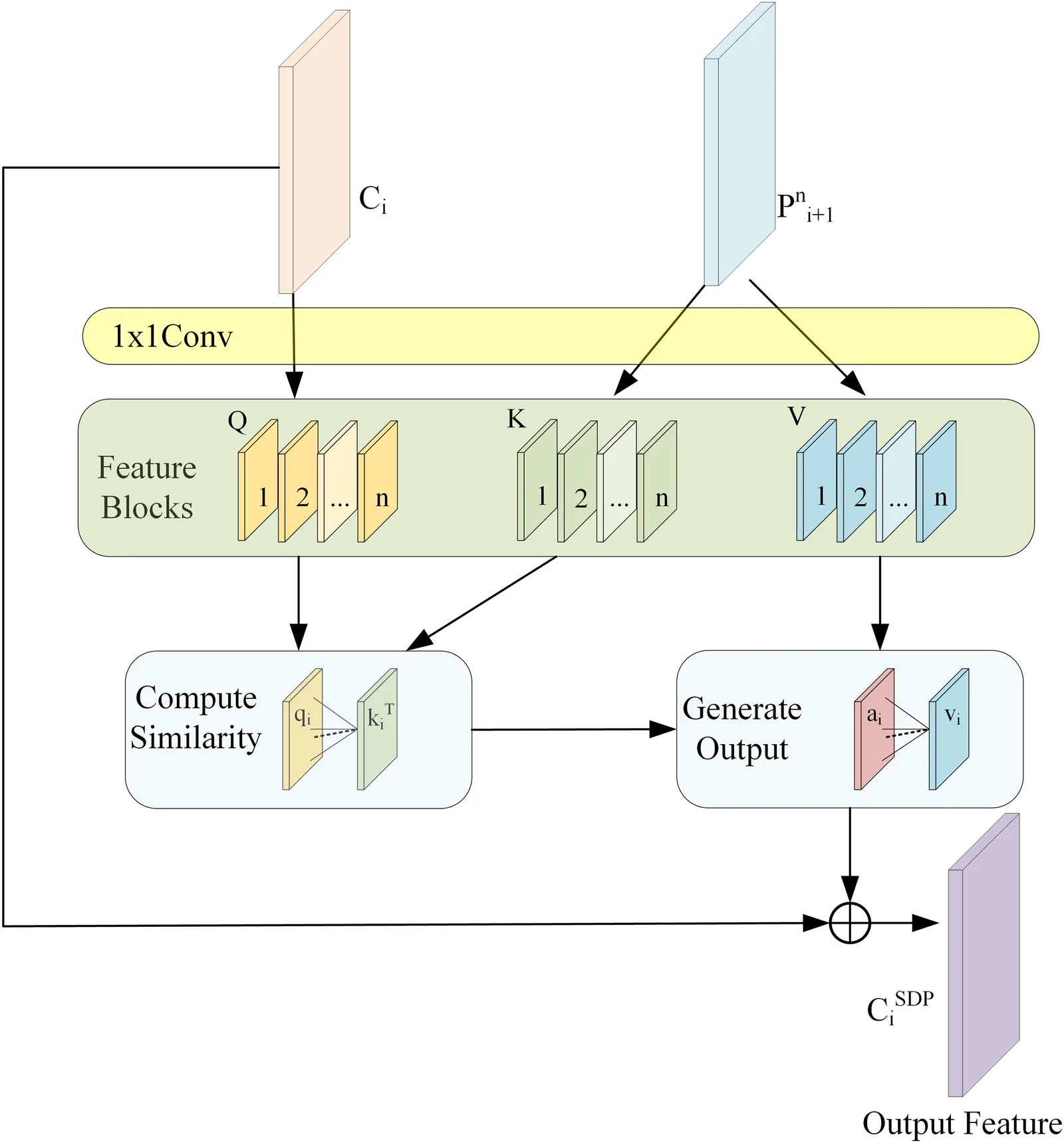
Architecture of the spatial dependency modeling module.

Inspired by ViT/Transformer [35], this paper introduces a spatial dependency modeling mechanism to characterize the correspondences between low-level detail features and high-level semantic features. Its architecture is shown in Fig 6.

First, the high-level feature $P_{i+1}$ is upsampled to the same spatial resolution as the low-level feature $C_{i}$, denoted as $P_{i+1}^{u}$. Subsequently, three sets of feature representations Q, K and V are generated through linear mappings (i.e., 1×1 convolutions), where the low-level features are used to construct the query representation and the high-level features provide semantic guidance information. Next, the features are divided into several local regions. Within each local region, spatial correlations are established by computing the correlation between low-level and high-level features, which can be expressed as:


$$\begin{array}{c} a_{i}=Softmax\;\left(\frac{q_{i}k_{i}^{T}}{\sqrt{c}}\right),\;i=1,2,...,n \end{array}$$ (8)

Here, $q_{i}$ and $k_{i}$ denote the query and key representations in the *i*th local region, $(\cdot {)}^{T}$ denotes transpose, *c* is the number of channels in the low-level feature $C_{i}$, and Softmax(·) normalizes the correlation weights. Based on these correlation weights, the high-level features within the corresponding region are adaptively combined through a weighted summation to generate the fused feature representation. This process enables the high-level semantic information to be adaptively adjusted according to the low-level structure, thereby enhancing the responses in key regions. Finally, each local region is reconstructed into a complete feature map and fused with the original low-level features to obtain the output feature $C_{i}^{SDP}$. By introducing this local spatial dependency modeling approach, the model is able to better preserve fine-grained details during multiscale feature interaction while incorporating high-level semantic supplementation, thereby improving the representation capability for tiny targets.

### RSA

**Fig 7:**
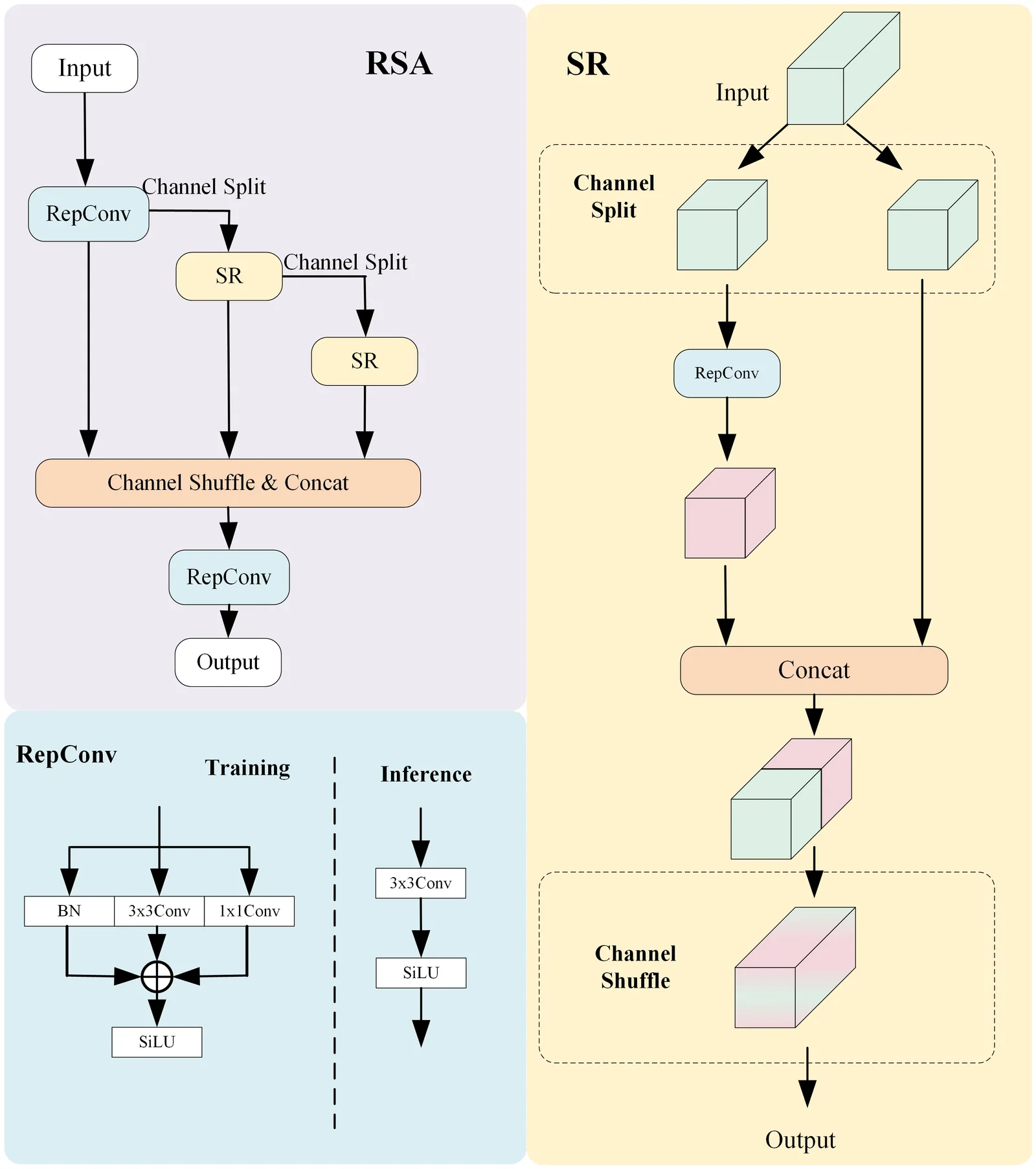
Architecture of the RSA module.

After the edge enhancement and smoothing denoising operations performed on the feature map by the Stem layer, we employ the RSA module to further improve feature extraction from the feature map. The architecture is shown in Fig 7.

#### (1) SR Module.

Inspired by the channel shuffling mechanism of ShuffleNet [36], this paper designs a feature extraction module that combines structural reparameterized convolution (RepConv) with channel shuffling, termed the SR (Shuffle RepConv) module. Assume the input feature map is $X\in {\mathbb{R}}^{C\times H\times W}$. First, it is evenly split along the channel dimension into two sub-feature tensors *X*_1_ and *X*_2_:


$$\begin{array}{c} X=\left[X_{1},X_{2}\right],\;X_{1},X_{2}\in {\mathbb{R}}^{\frac{C}{2}\times H\times W} \end{array}$$ (9)

Among them, the *X*_1_ branch directly performs identity mapping to preserve the original feature information, while the *X*_2_ branch undergoes feature extraction through the RepConv module. During the training phase, RepConv adopts a multibranch structure, including a 3×3 convolution branch, a 1×1 convolution branch, and an identity mapping branch, thereby enhancing the network’s representational capacity. During the inference phase, these branches are fused into a single 3×3 convolution through structural re-parameterization, which reduces inference computational overhead while maintaining representational capacity. After feature extraction, the output features of the two branches are concatenated along the channel dimension to obtain the output Y:


$$\begin{array}{c} Y=Concat\;\left(X_{1},RepConv\;\left(X_{2}\right)\right) \end{array}$$ (10)

Subsequently, a channel shuffling operation is applied to rearrange the feature channels, thereby promoting information interaction across different channels. Specifically, the features are rearranged according to a predefined number of groups. Through reshaping and transposition operations, the original channel grouping structure is broken, enabling cross-channel information fusion.

By introducing channel splitting and channel shuffling mechanisms, the SR module maintains sufficient feature representation capability. Meanwhile, the structural re-parameterization property of RepConv enables the model to learn richer feature representations during the training phase, while transforming into an efficient single-branch convolutional structure during the inference phase, thereby ensuring model inference efficiency.

#### (2) RSA Module.

Based on the SR module, this paper proposes the RSA module, which builds upon the One-Shot Aggregation (OSA) structure. The OSA module was originally introduced by VoVNet [37], with its core idea being to perform one-shot feature aggregation after multi-layer feature extraction, thereby avoiding the computational and storage overhead associated with the dense connections in DenseNet [38]. In the proposed RSA module architecture, RepConv is first used to perform channel compression and preliminary feature extraction on the input features. Subsequently, multiple SR modules are stacked to further extract features. Specifically, the SR modules are stacked in two stages:


$$\begin{array}{c} X_{1}=RepConv\;\left(X\right) \end{array}$$ (11)

$$\begin{array}{c} X_{2}={SR}_{1}\;\left(X_{1}\right),\;X_{3}={SR}_{2}\;\left(X_{2}\right) \end{array}$$ (12)

This stage-wise stacking structure gradually enhances feature representation capability while promoting information interaction across channels. Finally, the features from different stages are concatenated along the channel dimension and passed through a RepConv for feature fusion and channel adjustment to obtain the final output feature Y:


$$\begin{array}{c} Y=RepConv\;\left(Concat\;\left(X_{1},X_{2},X_{3}\right)\right) \end{array}$$ (13)

In addition, an optional SE attention mechanism can be introduced to further enhance the feature response capability along the channel dimension. Compared with the traditional OSA structure, RSA incorporates the SR module and structural reparameterized convolutions, enabling the network to achieve stronger feature modeling capability during the training phase while maintaining inference efficiency during the inference phase. In the multiscale feature fusion stage, it facilitates information interaction among features at different scales, thereby enhancing the semantic transmission capability between different levels of the feature pyramid and further improving object detection performance.

In summary, EES-Stem operates at the input stage to preserve edge cues while applying explicit smoothing, FSC-FPN acts in the neck to retain high-frequency detail and align cross-scale spatial information, and RSA-Block reorganizes and aggregates backbone features using established channel-shuffling, RepConv, and OSA principles. These established techniques are not presented as new individual operations; the contribution lies in their stage-specific adaptation and coordinated integration for SAR small-ship detection.

### Experimental results and analysis

This section first introduces the datasets used in the experiments, followed by a detailed description of the experimental settings, including the hardware and software environment configurations. On this basis, the evaluation metrics adopted in this paper are presented. Finally, the performance of the proposed method is evaluated through ablation experiments, comparative experiments, and scene-specific quantitative FP/FN analysis.

### Datasets

This paper uses the HRSID dataset as the primary experimental dataset and employs the SSDD dataset for auxiliary validation. The HRSID dataset contains 5,604 cropped SAR ship images with a total of 16,951 annotated targets, averaging approximately 3 ships per image, among which small, medium, and large ships account for 54.5%, 43.5%, and 2%, respectively. To comprehensively evaluate the model performance, the HRSID dataset is randomly divided into a training set (3,922 images), a validation set (561 images), and a test set (1,121 images) with a split ratio of 7:1:2, while maintaining the original distribution of ship sizes.

The SSDD dataset [39] contains a total of 1,160 SAR images and is similarly divided into a training set (820 images), a validation set (108 images), and a test set (232 images) with a split ratio of approximately 7:1:2. Both of the above datasets have been widely used in research on maritime ship and maritime object detection. The main characteristics of the datasets are summarized in Table 1.

**Table 1:** Parameters of the HRSID and SSDD datasets.

Parameters	HRSID	SSDD
Data Source	Sentinel-1B, TerraSAR-X, TanDem-X	RadarSat-2, TerraSAR-X, Sentinel-1
Image size/pixels	800×800	About 500×500
Number of train set images	3922	820
Number of val set images	561	108
Number of test set images	1121	232
Total number	16,951	2,456

### Experimental setup

The experiments were conducted on a computer running the Linux operating system. The main configurations and setting parameters related to the experiments are summarized in Table 2 and Table 3, respectively.

**Table 2:** Experimental platform configuration information.

Configuration	Version
Operating system	Linux
CPU	13th Gen Intel(R) Core(TM) i7-13700
GPU	NVIDIA GeForce RTX 3090
CUDA	12.8
cuDNN	9.10.2
Compiler	Python 3.10.19
Framework	PyTorch 2.9.0 + cu128

**Table 3:** Detailed parameters of the training model.

Component	Name/value
Epochs	200
Image Size	640×640
Batch size	8
Optimizer	SGD (lr0 = 0.01; momentum = 0.9)
LR schedule	Linear decay to 0.0001
Weight decay	0.0005
Warm-up	3 epochs
Workers	8
AMP	False

All tabulated results are arithmetic means over five runs using random seeds 42, 123, 202, 303, and 404.

The model was trained for 200 epochs using the Stochastic Gradient Descent (SGD) optimizer, with a batch size of 8, a momentum of 0.9, and a weight decay of 0.0005. The detection loss consists of a CIoU-based bounding-box regression loss, a binary cross-entropy classification loss, and Distribution Focal Loss (DFL) for bounding-box distribution regression. Early stopping was not used. The learning rate was linearly decayed from an initial value of 0.01 to 0.0001, with a warm-up period of three epochs. The main data augmentation strategies included hue–saturation–value (HSV) perturbation (*h* = 0.015, *s* = 0.7, and *v* = 0.4), translation (0.1), scaling (0.5), horizontal flipping (0.5), and mosaic augmentation (1.0). Mosaic augmentation was disabled during the final 10 epochs. Automatic mixed precision (AMP) was disabled. The remaining hyperparameter settings are summarized in Table 3.

### Evaluation metric

We adopt the COCO evaluation metrics to assess model performance, with the core metric being Average Precision (AP). In the COCO evaluation system, AP is defined as the mean average precision computed across multiple IoU thresholds ranging from 0.5 to 0.95 with a step size of 0.05. Here, IoU (Intersection over Union) is mathematically defined as follows:


$$\begin{array}{c} IoU=\frac{\left|B_{p}\cap B_{gt}\right|}{\left|B_{p}\cup B_{gt}\right|} \end{array}$$ (14)

where $B_{p}$ is the predicted box and $B_{gt}$ is the ground-truth box.

${AP}_{50}$ denotes the average precision at IoU=0.5, while ${AP}_{75}$ denotes the average precision at IoU=0.75, which imposes a more stringent requirement on target localization accuracy.

Precision (*P*), Recall (*R*), and mean Average Precision (mAP) are all calculated based on true positives (TP), false positives (FP), and false negatives (FN) in the model predictions. Specifically, precision measures the proportion of correctly predicted positive samples among all samples predicted as positive, while recall reflects the model’s ability to identify actual positive samples. Their mathematical definitions are as follows:


$$\begin{array}{c} P=\frac{N_{TP}}{N_{TP}+N_{FP}} \end{array}$$ (15)

$$\begin{array}{c} R=\frac{N_{TP}}{N_{TP}+N_{FN}} \end{array}$$ (16)

where $N_{TP}$, $N_{FP}$ and $N_{FN}$ represent the numbers of true positives, false positives, and false negatives, respectively.

**Fig 8:**
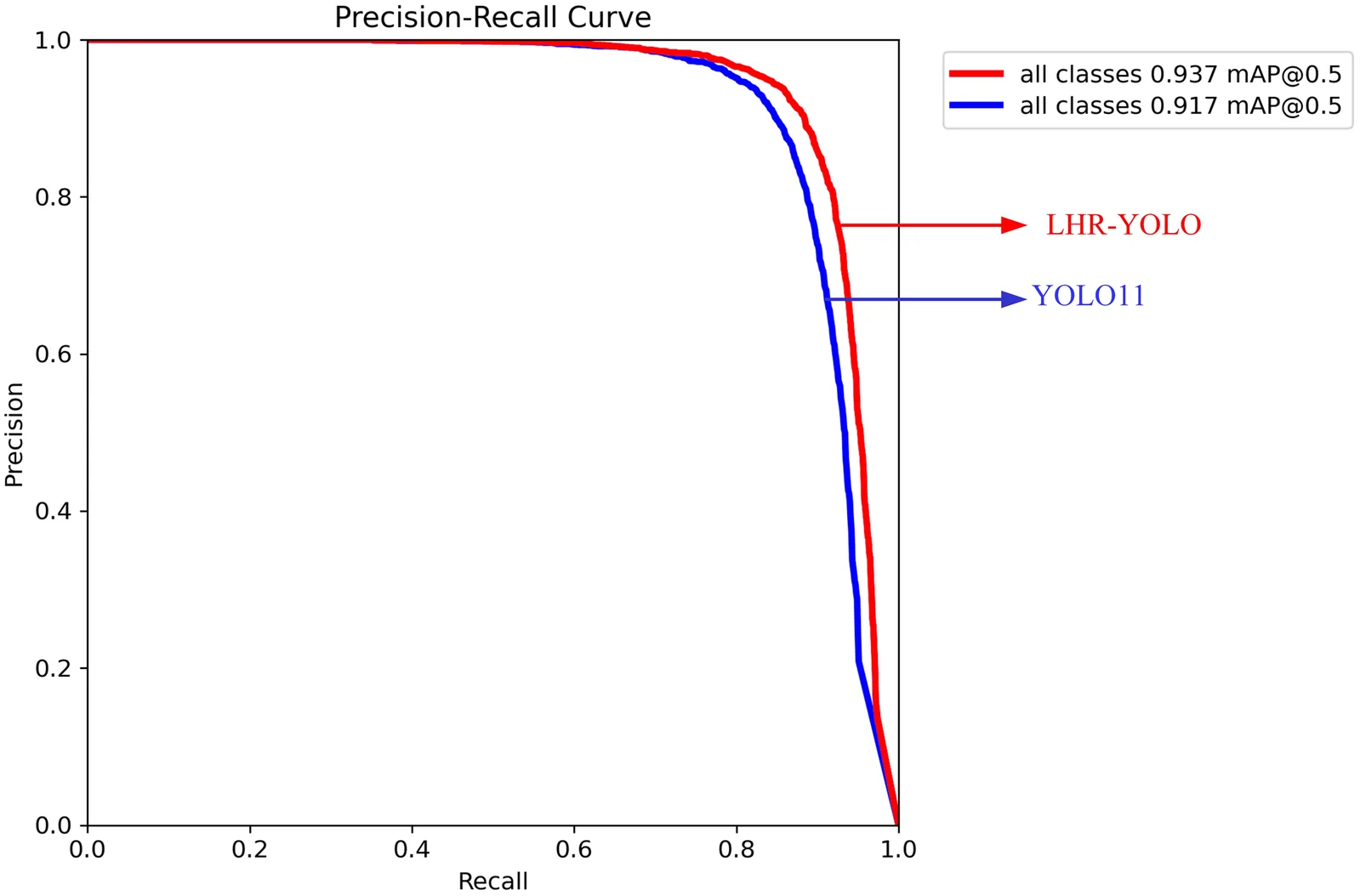
Comparison of PR curves between YOLO11 and LHR-YOLO. The blue curve represents YOLO11, and the red curve represents LHR-YOLO.

For the precision and recall metrics, Fig 8 shows the precision-recall curves for each category on the HRSID dataset, where the horizontal axis represents recall and the vertical axis represents precision. By comparison, it can be observed that the area enclosed by the P-R curve of YOLO11 and the axes is smaller than that of our improved algorithm model, indicating that under the same recall rate, our model achieves superior precision for these categories.

For each category *j*, the corresponding average precision is denoted as ${AP}_{j}$. The mean Average Precision (mAP) is defined as the average of all ${AP}_{j}$ values across all categories, where *N* represents the total number of categories. The mathematical definitions of AP and mAP are given as follows:


$$\begin{array}{c} AP=\int_{0}^{1}P(R)\;dR \end{array}$$ (17)

$$\begin{array}{c} mAP=\frac{1}{N}\sum_{j=1}^{N}{AP}_{j} \end{array}$$ (18)

When calculating AP and mAP, an IoU threshold must be set. We follow the COCO standard and use multiple IoU thresholds. The detailed definitions of these metrics are provided in Table 4.

**Table 4:** Definitions of evaluation metrics.

Metric	Meaning
*AP*_50:95_	AP For IoU = 0.5:0.05:0.95
*AP*_50_	AP at IoU = 0.50
*AP*_75_	AP at IoU = 0.75
$AP_{S}$	AP For small targets(IoU = 0.50:0.05:0.95)
$AP_{M}$	AP For medium targets(IoU = 0.50:0.05:0.95)
$AP_{L}$	AP For large targets(IoU = 0.50:0.05:0.95)

### Ablation study

To evaluate the contribution of each proposed module, we assessed EES-Stem, FSC-FPN, and RSA-Block both individually and in different combinations with the baseline model. The results show that the three modules improve different aspects of the detection performance, supporting their effectiveness as integrated architectural components.

First, introducing EES-Stem at the initial feature extraction stage improves the representation of target contours and local structural information. Under complex backgrounds and noise interference, the edge-aware design helps enhance target-boundary information and reduce the influence of irrelevant background responses. Specifically, ${mAP}_{50}$ increases from 91.11% to 91.29%, while the improvement is more pronounced under the stricter ${mAP}_{50:95}$ metric, which increases from 68.13% to 69.47%. These results indicate that EES-Stem contributes positively to localization accuracy, as shown in Table 5.

**Table 5:** Ablation study on the HRSID dataset using the main evaluation metrics. All values are reported as percentages.

Baseline	EES-Stem	FSC-FPN	RSA	${mAP}_{50}$	${mAP}_{75}$	${mAP}_{50:95}$
✓				91.11	77.65	68.13
✓	✓			91.29	79.18	69.47
✓		✓		92.93	80.67	69.65
✓			✓	91.77	78.71	69.02
✓	✓	✓		93.19	81.82	70.94
✓	✓		✓	91.90	80.09	70.13
✓		✓	✓	93.29	81.42	70.44
✓	✓	✓	✓	93.57	82.52	71.59

Second, incorporating high-frequency information enhancement and cross-layer spatial dependency modeling at the feature fusion stage improves the representation of small-scale targets. This design helps alleviate the weakening of small-target features during multiscale feature propagation. Specifically, ${mAP}_{S}$ increases from 53.95% to 58.22%, corresponding to a gain of 4.27 percentage points. This result demonstrates the contribution of FSC-FPN to small-target feature discrimination, as shown in Table 6.

**Table 6:** Ablation study on the HRSID dataset using scale-specific evaluation metrics. All values are reported as percentages.

Baseline	EES-Stem	FSC-FPN	RSA	${mAP}_{𝐒}$	${mAP}_{𝐌}$	${mAP}_{𝐋}$
✓				53.95	78.97	46.64
✓	✓			55.43	80.15	47.99
✓		✓		58.22	78.61	49.97
✓			✓	54.50	79.73	53.01
✓	✓	✓		59.76	79.75	50.03
✓	✓		✓	55.85	80.58	52.10
✓		✓	✓	58.94	79.26	50.35
✓	✓	✓	✓	60.53	80.10	49.10

Finally, introducing RSA-Block further improves feature representation through cross-channel interaction and multistage feature aggregation. Specifically, ${mAP}_{50}$, ${mAP}_{75}$, and ${mAP}_{50:95}$ increase from 91.11%, 77.65%, and 68.13% to 91.77%, 78.71%, and 69.02%, respectively, as shown in Table 5.

Overall, the module-level ablation study reveals the distinct contributions of the three proposed modules. EES-Stem primarily improves localization-sensitive metrics, increasing ${mAP}_{50:95}$ from 68.13% to 69.47%, corresponding to a gain of 1.34 percentage points. FSC-FPN provides the most pronounced improvement in small-target detection, increasing ${mAP}_{S}$ from 53.95% to 58.22%, a gain of 4.27 percentage points. When introduced individually, RSA improves the overall detection performance, with ${mAP}_{50}$, ${mAP}_{75}$, and ${mAP}_{50:95}$ increasing by 0.66, 1.06, and 0.89 percentage points, respectively. It also increases ${mAP}_{L}$ from 46.64% to 53.01%. Combining all three modules yields the highest overall ${mAP}_{50:95}$ and ${mAP}_{S}$, reaching 71.59% and 60.53%, respectively. However, the complete model does not achieve the highest ${mAP}_{L}$ among all configurations, indicating that the improvements are not uniform across different target scales. This observation is consistent with the primary design objective of improving small-target detection. Since the ablation experiments are conducted at the module level, the results support the effectiveness of EES-Stem, FSC-FPN, and RSA as integrated architectural components, but do not isolate the independent contribution of every internal operation.

### Comparisons with representative methods

To evaluate the effectiveness of the proposed method, comparative experiments were conducted on the HRSID dataset. Representative object detection methods were selected for comparison, including one-stage detectors such as YOLOv5, YOLOv6, YOLOv8, YOLOv9, YOLOv10, YOLO11, and YOLO26, as well as other widely used detectors, including ATSS, GFL, TOOD, and RTMDet.

To ensure a fair comparison, all models were implemented locally and trained from scratch under the same experimental protocol, using the same dataset split, input resolution, training schedule, and evaluation script. In particular, YOLO11n and YOLO26n refer to the locally implemented versions used in these experiments.

Table 7 presents the performance of the compared object detection methods on the HRSID dataset. In terms of overall detection performance, most YOLO-series models exhibit relatively stable results. For example, YOLOv5n, YOLOv8n, and YOLOv9t achieve ${mAP}_{50:95}$ values of 67.0%, 66.6%, and 66.8%, respectively. The baseline YOLO11n model further improves this metric to 68.1%, demonstrating favorable overall detection performance. By comparison, ATSS, TOOD, and RTMDet achieve ${mAP}_{50:95}$ values of 63.4%, 61.7%, and 62.5%, respectively, which are lower than those of most of the evaluated YOLO-series models.

**Table 7:** Comparison of different object detection methods on the HRSID dataset. All values are reported as percentages.

Method	${mAP}_{50:95}$	${mAP}_{50}$	${mAP}_{75}$	${mAP}_{𝐒}$	${mAP}_{𝐌}$	${mAP}_{𝐋}$
YOLOv5n [40] (2020)	67.0	90.3	77.2	55.1	78.6	54.2
ATSS [41] (2020)	63.4	89.9	72.0	50.9	75.8	53.0
GFL [42] (2020)	65.7	91.0	74.6	54.4	77.4	**56.2**
TOOD [43] (2021)	61.7	88.6	69.8	49.1	76.7	54.0
YOLOv6n [44] (2022)	65.2	89.5	74.1	52.0	78.1	41.1
YOLOv8n [45] (2023)	66.6	90.5	76.6	54.2	78.5	50.7
RTMDet [46] (2023)	62.5	88.1	71.9	49.6	75.7	53.6
YOLOv9t [47] (2024)	66.8	90.3	76.7	54.2	79.0	52.3
YOLOv10n [48] (2024)	66.8	89.9	77.2	54.5	78.6	48.4
YOLO11n (2024)	68.1	91.1	77.6	54.0	79.0	46.6
SIE-YOLO11 [49] (2025)	70.1	92.9	81.4	**60.5**	79.6	50.2
YOLO26n (2026)	67.5	91.6	77.2	54.5	77.7	46.0
LHR-YOLO (ours)	**71.6**	**93.6**	**82.5**	**60.5**	**80.1**	49.1

Compared with these methods, the proposed LHR-YOLO achieves improved performance on several key metrics. Specifically, LHR-YOLO reaches an ${mAP}_{50:95}$ of 71.6%, outperforming YOLOv5n, YOLOv8n, and YOLO11n by 4.6, 5.0, and 3.5 percentage points, respectively. These results demonstrate the effectiveness of the proposed method in improving overall detection accuracy.

For small-target detection, LHR-YOLO achieves an ${mAP}_{S}$ of 60.5%, exceeding YOLOv5n and YOLOv8n by 5.4 and 6.3 percentage points, respectively. This result indicates that the proposed method improves feature representation for small-scale targets and consequently enhances small-target detection performance. Overall, LHR-YOLO demonstrates competitive performance in terms of both overall detection accuracy and small-target detection capability, supporting the effectiveness of the proposed architectural improvements.

Table 8 presents the performance of different object detection methods on the SSDD dataset. In terms of overall detection performance, earlier YOLO models, including YOLOv5n, YOLOv6n, and YOLOv8n, generally achieve ${mAP}_{50:95}$ values of approximately 60%. With subsequent architectural improvements, YOLO11n and YOLO26n achieve substantially higher ${mAP}_{50:95}$ values of 72.4% and 71.6%, respectively, demonstrating stronger overall detection performance than earlier YOLO variants. Meanwhile, ATSS, GFL, TOOD, and RTMDet achieve results comparable to those of several earlier YOLO models on this dataset.

**Table 8:** Comparison of different object detection methods on the SSDD dataset. All values are reported as percentages.

Method	${mAP}_{50:95}$	${mAP}_{50}$	${mAP}_{75}$	${mAP}_{𝐒}$	${mAP}_{𝐌}$	${mAP}_{𝐋}$
YOLOv5n [40] (2020)	59.1	97.6	64.3	52.3	69.5	68.0
ATSS [41] (2020)	61.2	95.6	72.4	56.7	72.1	53.9
GFL [42] (2020)	59.5	93.2	71.6	55.3	70.6	53.5
TOOD [43] (2021)	60.7	95.8	70.5	57.1	70.4	51.0
YOLOv6n [44] (2022)	58.1	96.0	65.0	51.5	69.1	61.6
YOLOv8n [45] (2023)	60.6	97.0	68.9	53.9	70.9	65.6
RTMDet [46] (2023)	60.0	96.0	69.3	55.6	68.7	64.2
YOLOv9t [47] (2024)	60.1	96.6	66.6	53.3	69.9	68.9
YOLOv10n [48] (2024)	59.9	95.2	65.9	54.8	69.4	64.2
YOLO11n (2024)	72.4	**98.5**	87.3	68.3	74.8	70.9
SIE-YOLO11 [49] (2025)	62.7	97.9	72.2	56.0	72.5	64.2
YOLO26n (2026)	71.6	97.4	85.7	67.3	72.5	**71.1**
LHR-YOLO (ours)	**73.4**	98.4	**87.9**	**68.8**	**75.6**	68.6

The proposed LHR-YOLO further improves the overall detection performance, achieving an ${mAP}_{50:95}$ of 73.4%. This result exceeds those of YOLO11n and YOLO26n by 1.0 and 1.8 percentage points, respectively, demonstrating the effectiveness of the proposed architectural improvements.

For small-target detection, LHR-YOLO achieves an ${mAP}_{S}$ of 68.8%, exceeding YOLO11n and YOLO26n by 0.5 and 1.5 percentage points, respectively. This result indicates that the proposed method improves the preservation and representation of small-target features. Overall, LHR-YOLO achieves the highest ${mAP}_{50:95}$, ${mAP}_{75}$, ${mAP}_{S}$, and ${mAP}_{M}$ values on the SSDD dataset, demonstrating competitive overall detection performance and small-target detection capability.

**Fig 9:**
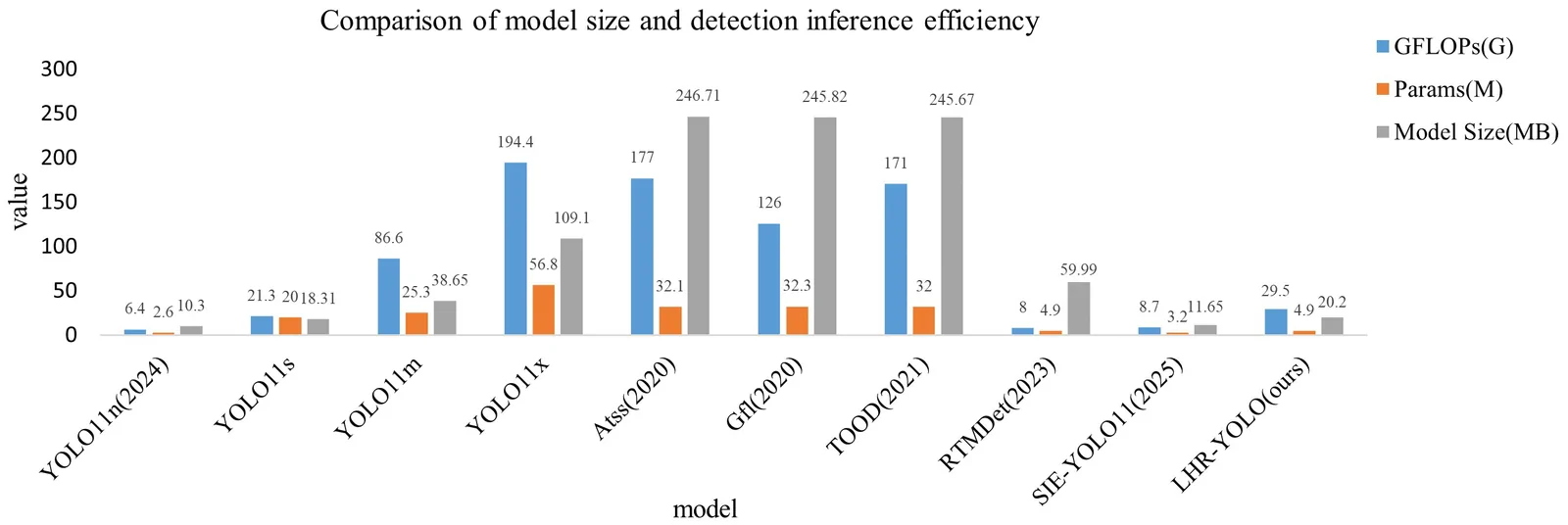
Comparison of model size and inference efficiency.

To evaluate model complexity and inference efficiency, we report the number of parameters, computational cost in GFLOPs, and model size. The corresponding results are presented in Fig 9. GFLOPs were calculated for a single forward pass during inference. Compared with the baseline YOLO11n model, LHR-YOLO introduces additional model complexity, with the number of parameters increasing from 2.6M to 4.9M. Nevertheless, it achieves improved detection accuracy. On the HRSID dataset, ${mAP}_{50:95}$ increases from 68.13% to 71.59%, corresponding to a gain of 3.46 percentage points, while ${mAP}_{S}$ increases from 53.95% to 60.53%, corresponding to a gain of 6.58 percentage points.

The additional computational cost mainly arises from the high-resolution detection head and the feature-enhancement operations introduced by EES-Stem, FSC-FPN, and RSA. The high-resolution branch preserves more spatial details but also increases feature-map computation and memory consumption. In addition, the proposed modules introduce filtering, cross-scale interaction, and feature aggregation operations. Therefore, the improvement in detection accuracy involves a trade-off in computational efficiency, and further lightweight optimization may be required for deployment in resource-constrained environments.

### Quantitative FP/FN analysis

To complement the aggregate detection metrics with scene-specific error statistics, we conducted a quantitative analysis of false positives (FPs) and false negatives (FNs) in nearshore, offshore, dense-target, and low-contrast scenes. All predictions were processed using non-maximum suppression with an IoU threshold of 0.70. The remaining detections were evaluated at a confidence threshold of 0.25, and a prediction was considered a true positive when its IoU with a ground-truth box was at least 0.50. The four scene groups were defined independently; therefore, the dense-target and low-contrast groups may overlap with the nearshore and offshore groups (Table 9).

**Table 9:** Quantitative FP/FN analysis for four challenging scene groups in the HRSID dataset. Values are means across five independent runs with different random seeds.

Scene	Model	FP/image	FN/image	Precision (%)	Recall (%)
Nearshore	YOLO11n	1.57	1.96	75.72	71.47
Nearshore	LHR-YOLO	2.20	1.31	71.61	80.84
Offshore	YOLO11n	0.10	0.06	95.08	96.71
Offshore	LHR-YOLO	0.12	0.04	93.94	98.11
Dense-target	YOLO11n	1.39	1.88	82.78	77.99
Dense-target	LHR-YOLO	1.96	1.25	78.85	85.39
Low-contrast	YOLO11n	0.94	1.28	77.95	72.30
Low-contrast	LHR-YOLO	1.32	0.90	73.79	80.56

At the evaluated confidence threshold, LHR-YOLO consistently reduces FN/image and increases recall across all four scene groups. However, it also increases FP/image and decreases precision. The improvement is therefore characterized by a recall–precision trade-off rather than a uniform reduction in all types of detection errors. The gains in recall are more pronounced in the nearshore, dense-target, and low-contrast groups, whereas the difference between the two models is relatively small in offshore scenes. These threshold-specific results characterize the distribution of detection errors under challenging scene conditions and complement the aggregate mAP results reported above.

## Conclusion

In this paper, we address key challenges in small-target detection within SAR images, including strong noise interference, blurred edge structures, and the vulnerability of small-target features to loss during multiscale transmission. Based on the YOLO11 framework, we propose an improved model, termed LHR-YOLO. From the perspective of feature modeling, systematic optimizations are introduced at critical stages of the network. At the input stage, an edge-aware EES-Stem is designed, which employs a Laplacian of Gaussian operator and Gaussian smoothing to enhance target boundary representation and mitigate noise interference. At the feature fusion stage, an FSC-FPN is constructed to achieve collaborative modeling of high-frequency information and spatial dependencies, effectively mitigating the attenuation of small-target features during cross-layer transmission. At the feature extraction stage, an RSA module is designed to enhance feature representation capability through channel interaction and multi-stage feature aggregation. These improvements synergistically enhance the model’s adaptability to complex SAR scenarios from three perspectives: input, fusion, and representation. Experimental results demonstrate that LHR-YOLO improves performance on both the HRSID and SSDD datasets, particularly for small-target detection. Module-level ablation studies provide evidence for the contributions of the three principal design units, while the scene-wise FP/FN analysis characterizes the error behavior of the complete model under four challenging conditions. The method also has limitations. Its high-resolution detection branch and feature-enhancement modules increase the parameter count and computational burden relative to YOLO11n, and the design and evaluation primarily target small ships. The complete model does not provide the best large-target result in the module-level ablation, and the scene groups used for the FP/FN analysis are derived from HRSID and may overlap. Future work will focus on finer component-level analysis, lightweight model design, and deployment optimization to enhance practicality while maintaining detection performance.
